# Differential Gene Expression in Foxtail Millet during Incompatible Interaction with *Uromyces setariae-italicae*

**DOI:** 10.1371/journal.pone.0123825

**Published:** 2015-04-17

**Authors:** Zhi Yong Li, Nan Wang, Li Dong, Hui Bai, Jian Zhang Quan, Lei Liu, Zhi-Ping Dong

**Affiliations:** 1 Department of plant protect, Millet Institute, Hebei Academy of Agricultural and Forestry Sciences, National Foxtail Millet Improvement Center, Minor Cereal Crops Laboratory of Hebei Province, Shijiazhuang, China; 2 College of Life Science, Hebei Normal University, Shijiazhuang, China; National Institute of Plant Genome Research (NIPGR), INDIA

## Abstract

Foxtail millet (*Setaria italica*) is an important food and fodder grain crop that is grown for human consumption. Production of this species is affected by several plant diseases, such as rust. The cultivar Shilixiang has been identified as resistant to the foxtail millet rust pathogen, *Uromyces setariae-italicae*. In order to identify signaling pathways and genes related to the plant’s defense mechanisms against rust, the Shilixiang cultivar was used to construct a digital gene expression (DGE) library during the interaction of foxtail millet with *U*. *setariae-italicae*. In this study, we determined the most abundant differentially expressed signaling pathways of up-regulated genes in foxtail millet and identified significantly up-regulated genes. Finally, quantitative real-time polymerase chain reaction (qRT-PCR) analysis was used to analyze the expression of nine selected genes, and the patterns observed agreed well with DGE analysis. Expression levels of the genes were also compared between a resistant cultivar Shilixiang and a susceptible cultivar Yugu-1, and the result indicated that expression level of Shilixiang is higher than that of Yugu-1. This study reveals the relatively comprehensive mechanisms of rust-responsive transcription in foxtail millet.

## Introduction

During the protracted course of plant-pathogen co-evolution, plants have evolved a series of complex mechanisms to protect themselves effectively against attack by pathogens [1]. Plants ward off pathogens primarily through innate immunity. Pattern recognition receptors (PRRs) on the surface of plant cell membranes recognize pathogen-associated molecular patterns (PAMPs), which results in pattern-triggered immunity (PTI) [2]. The interaction of PAMPs with PRRs likely activates a signal transduction cascade that may involve mitogen-activated protein kinase (MAPK), reactive oxygen species (ROS), nitric oxide (NO), and other signaling events [3, 4]. However, pathogens have in turn evolved a set of mechanism to suppress PTI by injecting effectors into host cells [5]. Hence, plants have also evolved corresponding resistance (*R*) proteins that recognize and inhibit specific pathogen effectors, a process called effector-triggered immunity (ETI) [6]. Plants also defend against pathogens through induced-defense responses, such as systemic acquired resistance (SAR), which can induce a long-lasting, broad-spectrum resistance to microorganisms [7]. SAR can induce a hypersensitive response (HR) at the site of infection, resulting in rapid cell death in infected tissues [8]. The induction of SAR is accompanied by accumulation of the signal molecule salicylic acid (SA) and expression of pathogenesis-related (PR) genes [9].

Studies about differential gene expression during interactions with rust fungi have been reported in a number of cereals, including wheat [10] and pearl millet [11]. Foxtail millet is a staple food adapted to arid and dry areas of China and India [12, 13]. A reference genome sequence for foxtail millet has been generated and a haplotype map of the foxtail millet genome has been constructed, making it an ideal model crop for studying systems biology of cereals and bioenergy grasses [14, 15]. However, gene expression has not been assessed in the interaction between foxtail millet and its rust pathogen, *Uromyces setariae-italicae*. This rust is one of the most important diseases of foxtail millet worldwide. The disease can be epidemic in the short term, and in severe cases can cause losses of ~10–30% of yields. Based on our screening appraisal, the foxtail millet cultivar Shilixiang has the best rust resistance among 16,800 millet germplasm resources collected from eight countries. Disease resistance is coherent in the seedling and adult stages, making it an ideal subject for research to reveal the mechanism of resistance to rust [16]. In recent years, studies of *U*. *setariae-italicae* have been limited mainly to basic biology [17]; the pathogen is a biotrophic airborne fungus that is hard to culture on fungi medium, which has made it difficult to study the genes involved in foxtail millet-rust interactions. Thus, a more global gene-expression approach should be useful for elucidating the molecular mechanisms of these interactions.

Compared to expression microarray technology, digital gene expression (DGE) profiling offers many advantages, including an unbiased view of the transcriptome, greater precision, simpler preprocessing, and consistent results compared to qPCR [18]. Thus, it is being used increasingly for transcriptome analysis as it generates a proportional digital output, enabling comparison across different samples [19]. It is a powerful tool that allows the generation of absolute rather than relative gene expression measurements and identification of specific genes [20]. Based on DGE technology, Qi et al. showed recently that the response to waterlogging by differentially expressed cucumber genes is mainly related to carbon metabolism, photosynthesis, ROS generation/scavenging, and hormone synthesis/signaling [21]. In this study, we identified foxtail millet metabolic pathways or signal transduction pathways and related genes that are transcriptionally regulated in response to *U*. *setariae-italicae* infection, using the DGE system on a whole-genome scale. Quantitative real-time polymerase chain reaction (qRT-PCR) analysis was also used to validate the expression patterns of some important genes.

## Materials and Methods

### Plant material and inoculation

The foxtail millet cultivar Shilixiang and Yugu-1, respectively, have been identified as resistant and susceptible to attack by *U*. *setariae-italicae* (S1 Fig). The Shilixiang cultivar of foxtail millet was used in all experiments, and the Yugu-1 cultivar was used for qRT-PCR analysis. Millet seeds were surface sterilized in a 1% NaClO solution, and rinsed at least three times with sterile, double-distilled water. They were then planted in pots containing sterile soil. Ten pots were established, with each holding five seedlings. Once the sixth or seventh leaves had expanded, plants were inoculated with *U*. *setariae-italicae* urediniospores (5 × 10^6^ spores / mL) and incubated for 48 h at 28°C with 95% relative humidity, and then maintained in a growth chamber at 28°C under a photoperiod of 14h light and 10 h dark with a light intensity of 6000 lux. Leaf samples were collected at 0, 24, and 48 h after inoculation. One leaf was collected per pot, so that, a total of ten leaves were collected at each inoculation time. The collection was repeated three times at each time point. Samples were immediately quick-frozen in liquid nitrogen and then stored at -80°C until total RNA was isolated. These leaf samples were used as starting material for the DGE and qRT-PCR analysis.

### DGE library construction and sequencing

Total RNA was isolated from the leaf samples taken at 0, 24, and 48 h post-inoculation. RNA was extracted using an RNAprep Pure Plant Kit (TIANGEN Biotech Co., Ltd, Beijing, China) according to the manufacturer’s instructions. The quality and concentration of total RNA was checked using a Nanodrop ND 1000 spectrophotometer (NanoDrop, Wilmington, DE), and 6 μg of total RNA from the Shilixiang cultivar was used for Illumina sequencing.

Samples for establishing DGE libraries were prepared using an Illumina Gene Expression Sample Prep Kit. As described by Tang *et al*, tag libraries were constructed for the three different inoculation time samples (0 h, 24 h, and 48 h) [22]. After 15 cycles of linear PCR amplification, 95 bp fragments were purified by 6% TBE PAGE Gel electrophoresis. After denaturation, the single-chain molecules were fixed onto the Illumina Sequencing Chip (flowcell). Each molecule grows into a single-molecule cluster sequencing template through Situ amplification. Tag libraries were deep-sequenced using the Illumina HiSeq 2000 System. Four types of nucleotides that were labeled with four colors were added in, and we then performed sequencing by synthesis (SBS). Each tunnel generated millions of raw reads with a sequence length of 35 bp. Raw reads of the samples were deposited in the Sequence Read Archive (SRA) database of NCBI (accession number: SRP044723).

### Analysis and mapping of DGE tags

Raw image data obtained by sequencing was transformed by base calling into sequence data, which is called raw data or raw reads. Raw sequences have 3' adaptor fragments, some low-quality sequences, and several types of impurities. Raw sequences were transformed into Clean Tags by removing 3' adaptor sequences, empty reads (reads with only 3' adaptor sequences but no tags), low quality tags (those with unknown sequences 'N'), tags that were too long or too short, and those with a copy number of one (probably due to sequencing error). Clean tags were 21 nt long.

For gene expression annotation, we used a virtual library containing all the possible CATG+17 base length sequences of the reference gene sequences. All clean tags were mapped to the reference sequences, and only 1 bp mismatch was considered. Clean tags mapped to reference sequences from multiple genes were filtered, and any remaining clean tags were designated as unambiguous clean tags. The number of unambiguous clean tags for each gene was calculated and then normalized to TPM (number of transcripts per million clean tags). A rigorous algorithm was used to identify differentially expressed genes (DEGs) between every pair of samples. Foxtail millet genome sequence available at Phytozome (http://www.phytozome.net) was used as a reference to align the DEGs. According to the ascending order of physical position, DEGs were located individually on the foxtail millet chromosomes. Homology searches of DEGs were performed using the BLAST program against the non-redundant (nr) protein database in GenBank. GO enrichment analysis was conducted to look for significantly enriched GO terms in DEGs. Pathway enrichment analysis was used to identify significantly enriched metabolic pathways or signal transduction pathways in DEGs, compared to the whole genome background.

### Differential expression analysis using quantitative RT-PCR

As described above, total RNAs were extracted from leaf samples of the Shilixiang and Yugu-1 cultivars and their concentrations were determined. cDNA were synthesized using a Thermo Scientific RevertAid First Strand cDNA Synthesis Kit according to the manufacturer’s instructions (Thermo Fisher Scientific Inc., USA). The products of first strand cDNA synthesis were ready to be used directly in qPCR. PCR primers were designed using Primer Premier 5.0 software and synthesized by Sangon Biotech Co., Ltd. (Shanghai, China). The 18S rRNA gene (forward primer: 5’- AAAGTTGGGGGCTCGAAGAC -3’; reverse primer: 5’- GGTCGGCATCG TTTATGGTTG -3’) was used as an internal control to normalize the amount of gene-specific qRT-PCR products; primers used in the reactions are listed in S1 Table. Gene expression was quantified using an AB StepOne Real-Time PCR System. The Q-PCR system with SYBR Green detection was optimized for 20 μL reactions containing 10 μL 2× SYBR Premix (TaKaRa, Dalian, China), 0.4 μL ROX Reference Dye, 0.8 μL of each primer (10 μM), 2 μL cDNA template (100 ng/μL), and 6 μL nuclease-free water, according to the manufacturer’s guidelines. Control reactions contained the same mixtures with 2 μL of sterile water replacing the DNA template. Amplification of all samples was based on the following conditions: 95°C for 30 s, and 40 cycles of 95°C for 15 s and 60°C for 30 s. After each run, a melting curve of the product was generated to ensure specific amplification by increasing the temperature to 95°C for 15 s, cooling it to 60°C for 1 min, and then increasing it by 0.3°C/s to 95°C for 15 s with monitoring of fluorescence. Three technical replicates were performed per sample, and the average threshold cycle (CT) was calculated. Relative gene expression levels were calculated using the Applied Biosystems StepOne Software v2.0 (Applied Biosystems, Foster City, CA, USA) using the comparative 2^-ΔΔCT^ method [23].

## Results and Discussion

### Digital gene expression (DGE) library sequencing and mapping

**Fig 1:**
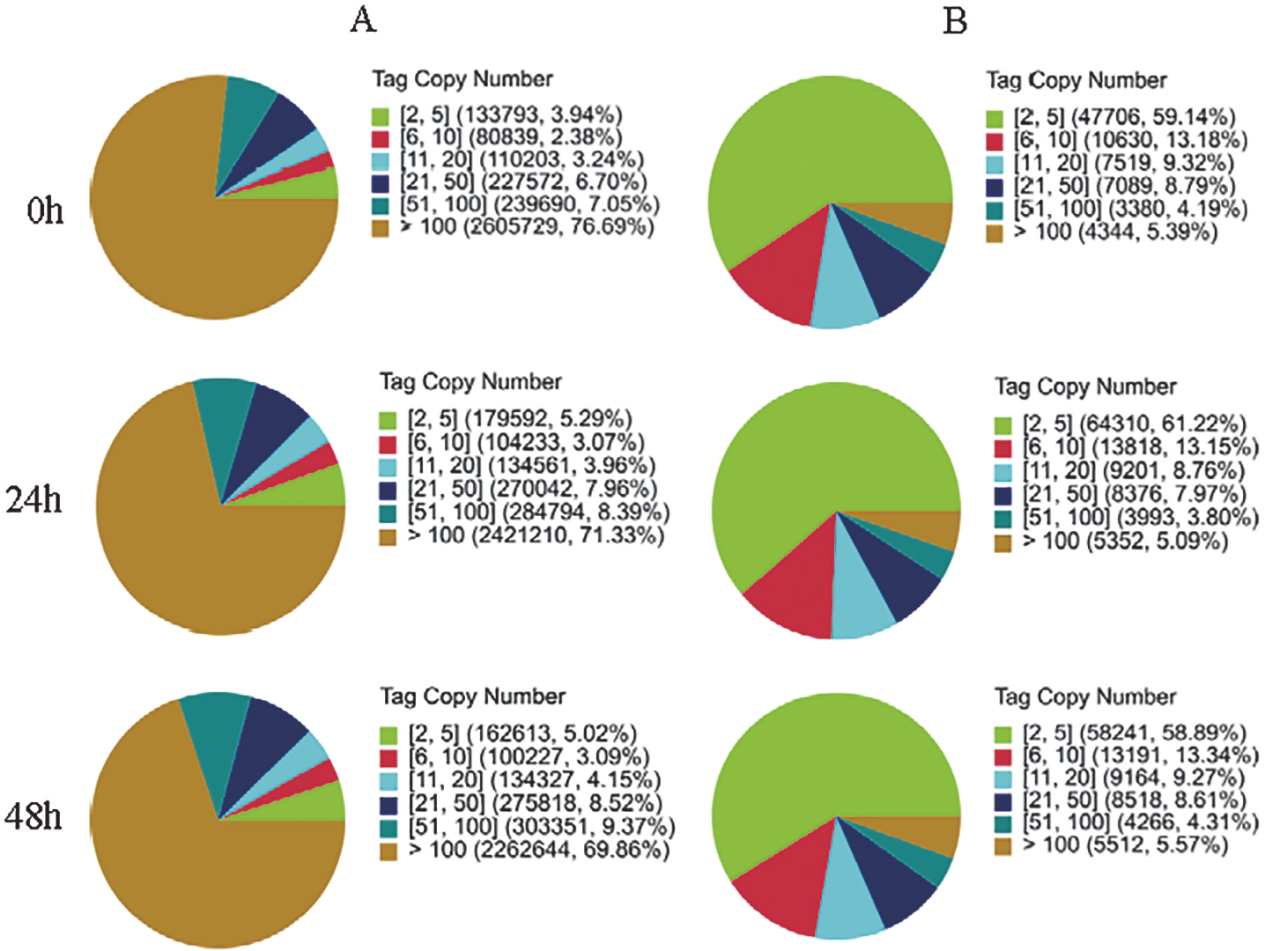
Distribution of total clean tags and distinct clean tags in each sample. The numbers in square brackets indicate the range of copy numbers of each tag category. The data in parentheses indicate the percentage of corresponding tags among the total clean tags and distinct clean tags. (A) Distribution of total clean tags. (B) Distribution of distinct clean tags.

Solexa/Illumina DGE analysis was performed to obtain a global view of the foxtail millet transcriptome after plants were inoculated with rust. Three Shilixiang millet DGE libraries were sequenced: at 0 h after inoculation with rust (SRX659703), at 24 h after inoculation (SRX659704), and at 48 h after inoculation (SRX659705)—this generated approximately three million raw tags in each library. After removing the low quality tags, the total number of clean tags for each of the three libraries ranged from 3.2 to 3.3 million and the number of tag entities with unique nucleotide sequences ranged from 80,668 to 105,050 (S2 Table). A foxtail millet Shilixiang leaf transcriptome reference gene database that included 32538 sequences (SRX800775, GBYO00000000) was preprocessed for tag mapping. Genes with a CATG site accounted for 82.88% of sequences. To obtain the reference tags, all the CATG+17 tags in the gene were taken as gene reference tags. Finally, 113,479 total reference tag sequences with 113,084 unambiguous reference tags were obtained. Among the clean tags, the number of sequences that could be mapped to unigenes ranged from 2.02 to 2.08 million, and the percentage of these clean tags ranged from 60.30 to 62.50% among the three libraries (S2 Table). To evaluate the DGE data, the distribution of the expression of clean tags were analyzed (Fig 1). The distribution of total clean tags and distinct clean tags across different tag abundance categories showed similar patterns for all three DGE libraries. In each library, the highly expressed tags dominated the total clean tags, but their distributions of distinct clean tags were small. In contrast, tags with a low level of expression among the total clean tags represented the majority of distinct clean tags. Results suggested that sequencing quality was high enough to enable the following analyses. Deep sequencing of foxtail millet during its interaction with *U*. *setariae-italicae* could facilitate the identification of signaling pathways and genes related to millet defense against rust.

### Variation in gene expression among different inoculation stages

**Fig 2:**
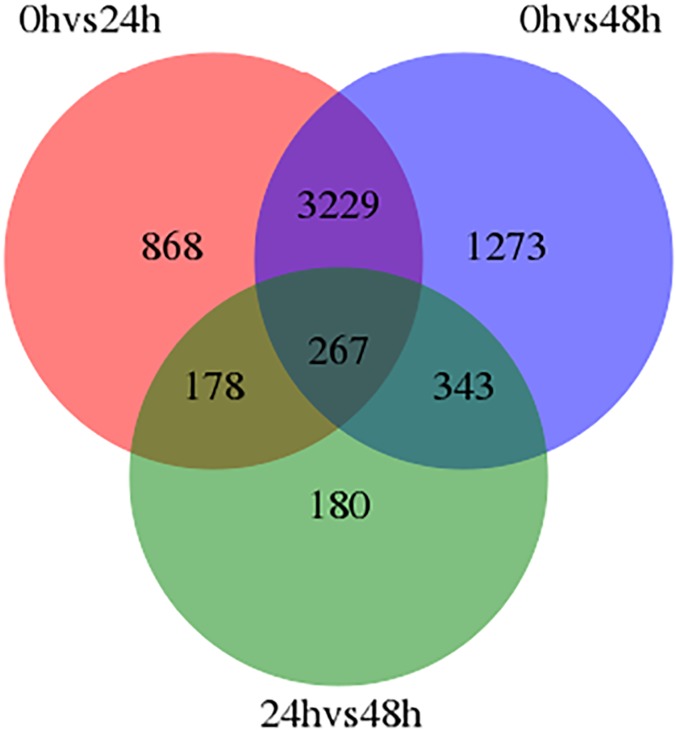
Venn diagram showing all of the DEGs.

**Fig 3:**
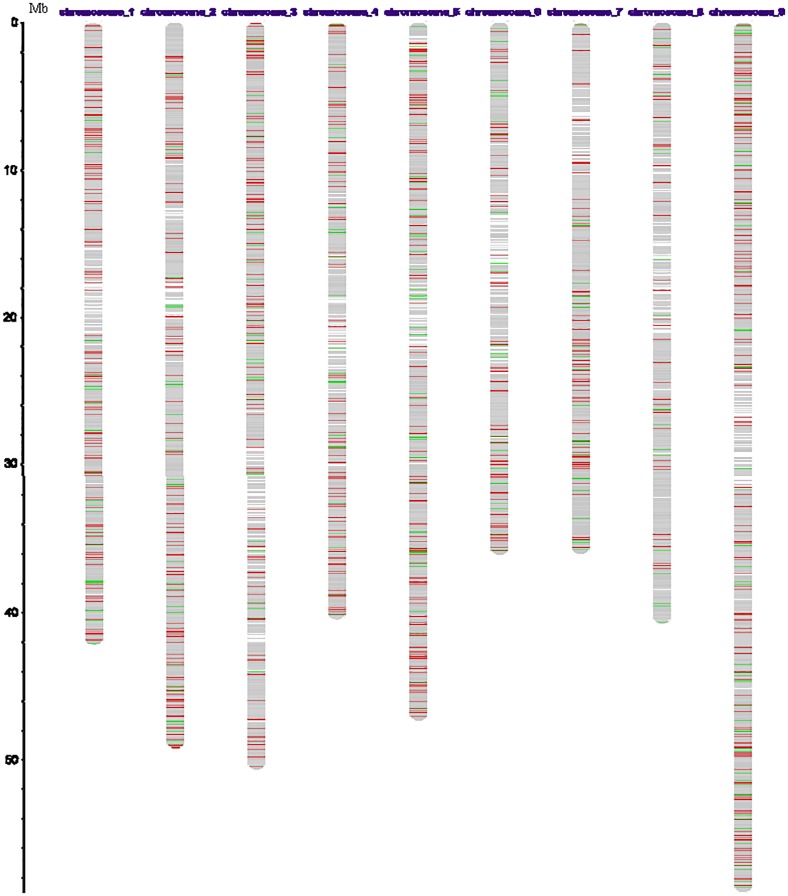
A physical map of foxtail millet 48h post-inoculation based on 5090 DEGs. Red lines represent up-regulated genes and green lines represent down-regulated genes.

To specifically identify genes related to rust inoculation in foxtail millet, the DEGs between two samples were identified. Comparison of DEGs in Shilixiang at 24 h after inoculation with rust versus Shilixiang inoculated with rust 0 h showed that 4542 genes differed significantly (FDR < 0.001, |log2 ratio|⩾1) between the two samples. Among them, 3442 genes were up-regulated and 1100 genes were down-regulated. Between Shilixiang at 48 h after inoculation with rust and Shilixiang inoculated with rust 0 h, a total of 5112 DEGs were detected, including both up-regulated (3941) and down-regulated genes (1171). We identified 968 genes that were expressed differently at 48 h post-inoculation compared to after only 24 h. Of those, 518 were up-regulated and 450 were down-regulated. A Venn diagram illustrating all of the DEGs is shown in Fig 2. A total of 267 genes were common to the 3 comparison groups, indicating that these genes are in a constant process of change. DEGs were also located on the chromosomes of foxtail millet, and physical maps were constructed; 5090 DEGs at 48 h post-inoculation were mapped to the nine chromosomes of foxtail millet (Fig 3). Among them, chromosome 9 had the most DEGs (961), followed by chromosome 5 (778), while chromosome 8 had the fewest (219; Table 1).

**Table 1:** Summary of the DEGs and their location on the nine chromosomes of foxtail millet 48h post-inoculation.

Chr1	Chr2	Chr3	Chr4	Chr5	Chr6	Chr7	Chr8	Chr9	Total
621	653	624	411	778	362	461	219	961	5090

### Gene Ontology functional enrichment analysis for DEGs

Gene ontology (GO) analysis of differentially expressed genes provided comprehensive evaluation of transcriptional variation in molecular functions, biological processes and cellular components. The numbers and assortment of the allocated GO categories provide a good indication of the diversity of the genes. A total of 3415, 3828 and 723 DEGs were categorized into the three main GO categories of three comparison groups (S2 Fig). And GO functional enrichment analysis (corrected P-value≤0.05) for DEGs was listed in S3 Table. For the DEGs, we found that ‘ribonucleoprotein complex’ and ‘macromolecular complex’ were the most significantly affected GO terms of cellular component; ‘structural molecule activity’ and ‘organic substance metabolic process’ terms were the most significantly affected GO terms of molecular function; and ‘cellular amino acid metabolic process’ was the most significantly affected GO term of biological process.

### KEGG analysis of metabolic pathways

Pathway enrichment analysis was used to identify significantly enriched metabolic pathways or signal transduction pathways in DEGs by comparing them with the whole genome background. Among all the genes with KEGG pathway annotation, 1121 differentially expressed genes were identified in the libraries between 0 and 24 h, 1258 differentially expressed genes between 0 and 48 h, and 222 differentially expressed genes between 24 and 48 h after inoculation. Among the top-10 most abundant differentially expressed signaling pathways of up-regulated millet genes, from every comparison group, the cluster for Ribosome represented the largest group (Table 2). In order of decreasing group size, this was followed by clusters for: Metabolic pathways; Biosynthesis of phenylpropanoids; Phenylalanine, tyrosine and tryptophan biosynthesis; Arginine and proline metabolism; Biosynthesis of alkaloids derived from shikimate pathway; Steroid biosynthesis; Phenylpropanoid biosynthesis; Flavonoid biosynthesis; Stilbenoid, diarylheptanoid and gingerol biosynthesis; Metabolism of xenobiotics by cytochrome P450; Valine, leucine and isoleucine biosynthesis; Fructose and mannose metabolism; Vitamin B6 metabolism; Benzoxazinoid biosynthesis; Taurine and hypotaurine metabolism; Fatty acid elongation in mitochondria; Glyoxylate and dicarboxylate metabolism; and Mismatch repair.

**Table 2:** Abundant differentially expressed signaling pathways of up-regulated genes in foxtail millet following interaction with the rust fungus *Uromyces setariae-italicae*.

**0h vs. 24h**	**Pathway**	**DEGs with pathway annotation (1121)**	**All genes with pathway annotation (10430)**	**Q value**	**Pathway ID**
1	Ribosome	102 (9.1%)	690 (6.62%)	1.591874e-02	ko03010
2	Biosynthesis of phenylpropanoids	73 (6.51%)	503 (4.82%)	9.801425e-02	ko01061
3	Biosynthesis of alkaloids derived from shikimate pathway	54 (4.82%)	355 (3.4%)	9.801425e-02	ko01063
4	Phenylalanine, tyrosine and tryptophan biosynthesis	16 (1.43%)	78 (0.75%)	1.129080e-01	ko00400
5	Steroid biosynthesis	9 (0.8%)	36 (0.35%)	1.515227e-01	ko00100
6	Arginine and proline metabolism	18 (1.61%)	98 (0.94%)	1.807406e-01	ko00330
7	Metabolism of xenobiotics by cytochrome P450	18 (1.61%)	104 (1%)	2.668737e-01	ko00980
8	Valine, leucine and isoleucine biosynthesis	10 (0.89%)	48 (0.46%)	2.668737e-01	ko00290
9	Vitamin B6 metabolism	4 (0.36%)	12 (0.12%)	2.668737e-01	ko00750
10	Metabolic pathways	293 (26.14%)	2489 (23.86%)	2.668737e-01	ko01100
**0h vs. 48h**	**Pathway**	**DEGs with pathway annotation (1258)**	**All genes with pathway annotation (10430)**	**Q value**	**Pathway ID**
1	Ribosome	120 (9.54%)	690 (6.62%)	5.609921e-04	ko03010
2	Arginine and proline metabolism	22 (1.75%)	98 (0.94%)	5.239005e-02	ko00330
3	Steroid biosynthesis	10 (0.79%)	36 (0.35%)	1.410173e-01	ko00100
4	Biosynthesis of phenylpropanoids	78 (6.2%)	503 (4.82%)	1.593453e-01	ko01061
5	Phenylalanine, tyrosine and tryptophan biosynthesis	16 (1.27%)	78 (0.75%)	2.798326e-01	ko00400
6	Metabolic pathways	329 (26.15%)	2489 (23.86%)	2.798326e-01	ko01100
7	Fructose and mannose metabolism	19 (1.51%)	101 (0.97%)	3.308341e-01	ko00051
8	Benzoxazinoid biosynthesis	10 (0.79%)	44 (0.42%)	3.308341e-01	ko00402
9	Taurine and hypotaurine metabolism	3 (0.24%)	7 (0.07%)	3.490632e-01	ko00430
10	Phenylpropanoid biosynthesis	41 (3.26%)	260 (2.49%)	3.490632e-01	ko00940
**24h vs. 48h**	**Pathway**	**DEGs with pathway annotation (222)**	**All genes with pathway annotation (10430)**	**Q value**	**Pathway ID**
1	Metabolic pathways	81 (36.49%)	2489 (23.86%)	4.209581e-04	ko01100
2	Flavonoid biosynthesis	9 (4.05%)	124 (1.19%)	2.959463e-02	ko00941
3	Biosynthesis of phenylpropanoids	18 (8.11%)	503 (4.82%)	2.443969e-01	ko01061
4	Steroid biosynthesis	3 (1.35%)	36 (0.35%)	4.103692e-01	ko00100
5	Biosynthesis of alkaloids derived from shikimate pathway	12 (5.41%)	355 (3.4%)	6.987740e-01	ko01063
6	Stilbenoid, diarylheptanoid and gingerol biosynthesis	6 (2.7%)	146 (1.4%)	7.249746e-01	ko00945
7	Fatty acid elongation in mitochondria	1 (0.45%)	5 (0.05%)	7.249746e-01	ko00062
8	Phenylpropanoid biosynthesis	9 (4.05%)	260 (2.49%)	7.249746e-01	ko00940
9	Glyoxylate and dicarboxylate metabolism	3 (1.35%)	59 (0.57%)	7.536070e-01	ko00630
10	Mismatch repair	2 (0.9%)	32 (0.31%)	7.536070e-01	ko03430

### Identification of defense-related genes in foxtail millet

Through pathway enrichment analysis in DGE, defense-related genes were discovered in foxtail millet. Some of the significantly up-regulated genes were described (S4 Table). They included 15 genes involved in the plant-pathogen interaction, 8 genes involved in the biosynthesis of phenylpropanoids and phenylpropanoid biosynthesis, and 2 genes involved in metabolism of xenobiotics by cytochrome P450 and glutathione metabolism. They also included a single gene involved in each of these categories: starch and sucrose metabolism; amino sugar and nucleotide sugar metabolism; aminoacyl-tRNA biosynthesis; natural killer-cell-mediated cytotoxicity; ribosome; terpenoid backbone biosynthesis; and finally biosynthesis of terpenoids and steroids. In addition to genes with clear function, novel genes with unknown functions were also found; they may be involved in rust resistance of foxtail millet, and their functions remain to be explored. This finding suggests that rust resistance in foxtail millet may include many unique processes and pathways.

PR proteins have been reported to be induced by pathogen infection, and they are known to have antifungal activity and enzymatic or inhibitory activity that improves the defensive abilities of plants [24, 25]. In the study, a significant increase in the PR genes were observed, such as PR1 (protein of unknown function), PR2 (β-1,3-glucanase), PR4 (endochitinase), and PR9 (peroxidase and peroxidase 1). Among them, PR1, β-1,3-glucanase, and endochitinase were mainly induced at 24 h post-inoculation, although their expression was still detected at 48 h, whereas the expression of peroxidase and peroxidase 1 showed higher transcript levels at 48 h of interaction (S4 Table). It is possible that β-1,3-glucanase and endochitinase hydrolyze β-1,3-glucanes and chitin, which are essential constituents of fungal cell walls that prevent further invasion of *U*. *setariae-italicae*. Peroxidase is an oxidative enzyme and it may be involved in defense reactions of foxtail millet against the rust pathogen at later stages of attack. Thus it might be possible that cooperation of PR proteins improves the ability of foxtail millet to fight against *U*. *setariae-italicae* infection. Expression of PR genes indicates the activation of SAR, and PR1 proteins have been used as markers for SAR [26]. Hence, the induction and up-regulation of PR proteins during the millet-rust interaction suggested that a SAR response had been induced.

**Fig 4:**
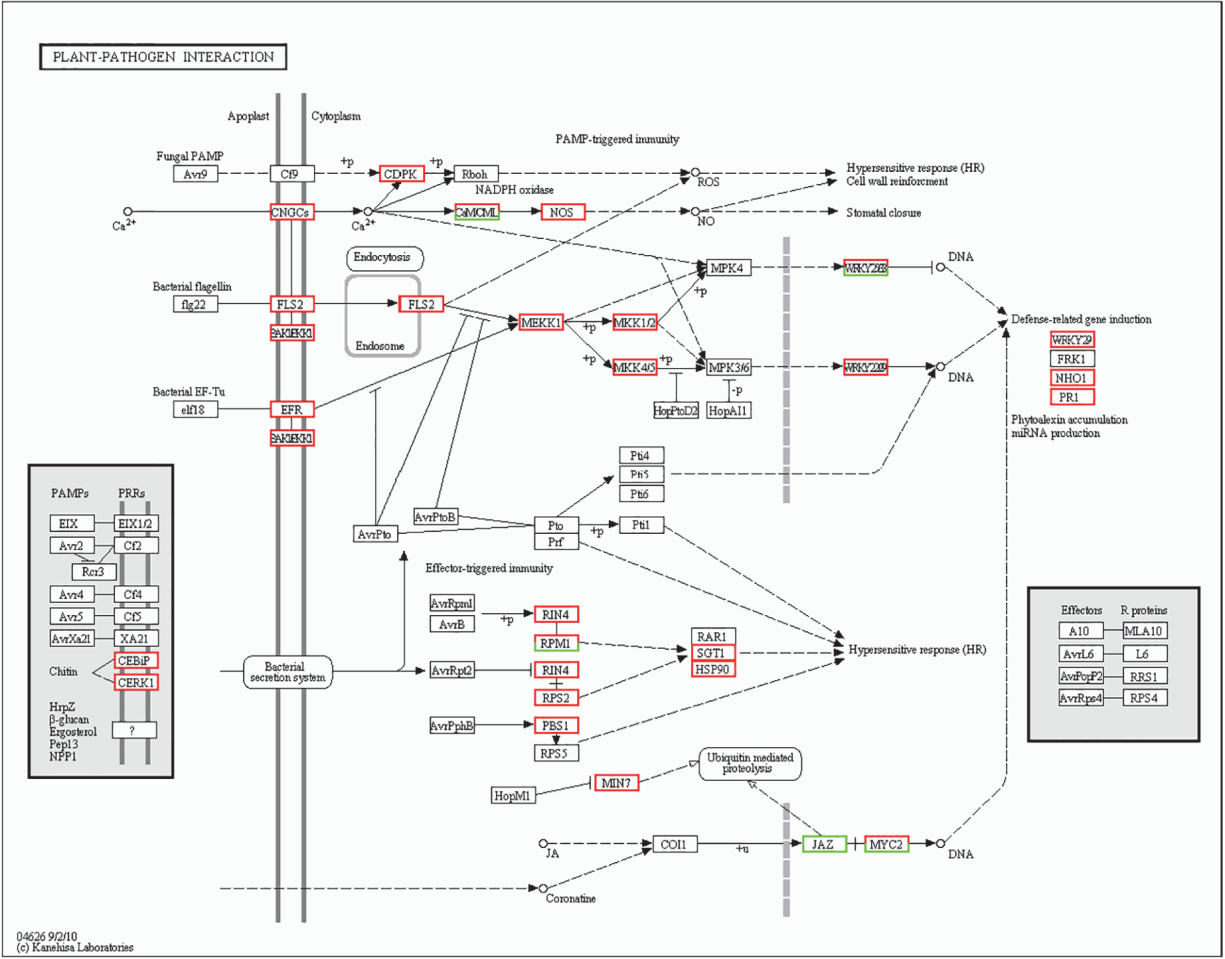
Change in gene expression of the plant-pathogen interaction pathway in foxtail millet 48h post-inoculation. Genes that up-regulated are marked with red borders while Genes that down-regulated are marked with green borders. Genes that did not change are marked with black borders.

Protein kinases are known to be involved directly in plant resistance; they play a central role in signaling during pathogen recognition and the subsequent activation of plant defense mechanisms. We identified four kinds of protein kinases—serine/threonine protein kinase, receptor-like kinase (RLK), calcium-dependent protein kinases (CDPKs), and mitogen-activated protein kinases (MAPKs)—that were up-regulated in the millet-rust plant-pathogen interactions. CDPK has been reported to trigger the production of ROS in response to pathogen infection in potato and tobacco [27, 28]. In the millet-rust interaction, NADPH oxidase *Rboh* was phosphorylated by CDPK, which was activated by elicitor-induced calcium influx. Co-infiltration of CDPK with *Rboh* led to the rapid production of ROS, which was accompanied by HR and resulted in resistance to the fungus (Fig 4). Cai et al. isolated and functionally analyzed a MAPKK gene *ZmMKK1* in maize group A, and found that the expression patterns of *ZmMKK1* were triggered by pathogen attack [29]. In our study, DGE analysis indicated that MAPKs of foxtail millet were up-regulated during interaction with *U*. *setariae-italicae*. Hence, we speculated that attack by the pathogen may have activated MEKK1-MKK1/2-MPK4 and MEKK1-MKK4/5-MPK3/6 cascades in the plants. WRKY proteins are among the identified substrates of the pathogen-responsive MAP-kinase-signaling cascades, and might function as a component in the MAP-kinases-signaling pathway involved in pathogen-induced HR [30, 31]. WRKY transcription factor, such us WRKY70 and WRKY60, were generally up-regulated in this study. WRKY proteins might be phosphorylated by MPK4 and MPK3/6, enhancing their DNA-binding and transcription activating activities. WRKY transcription factors may then activate WRKY-regulated genes, particularly defense-related genes, to fend off the invasion of *U*. *setariae-italicae* (Fig 4).

Disease resistance (R) genes in plants encode R proteins, which act as primary receptors of pathogen effector proteins or play indirect roles in this process [32]. In foxtail millet, we noted that two NBS-LRR disease resistance genes (RPM1 and RPS2) were up-regulated during interaction with the rust fungus. RPM1 recognizes AvrB and AvrRpm1, and RPS2 recognizes AvrRpt2. Yet during the RPM1 recognition process, a third protein, RIN4, is specifically required for RPM1 to function. It may participate in pathogen recognition complexes involving RPM1 [32]. Some signaling components, such as EDS1, NDR1, PBS3, RAR1 and SGT1, are required for resistance conferred by R genes, and they pass to the downstream of pathogen perception. In our study, SGT1 and NPR1 were up-regulated significantly (S3 Table). Austin et al. reported that mutation of *SGTlb* (one of two highly homologous *Arabidopsis* SGTl genes) disabled early plant defenses that are conferred by multiple R genes [33]. Therefore, we speculate that one of the related downstream signaling components of RPM1 and RPS2 might be SGT1. SGT1 and RAR1 might form the RARl-SGT1 complex [34]. Ultimately, rapid, localized programmed cell death (PCD) resulted from HR induced in plant cells (Fig 4).

**Fig 5:**
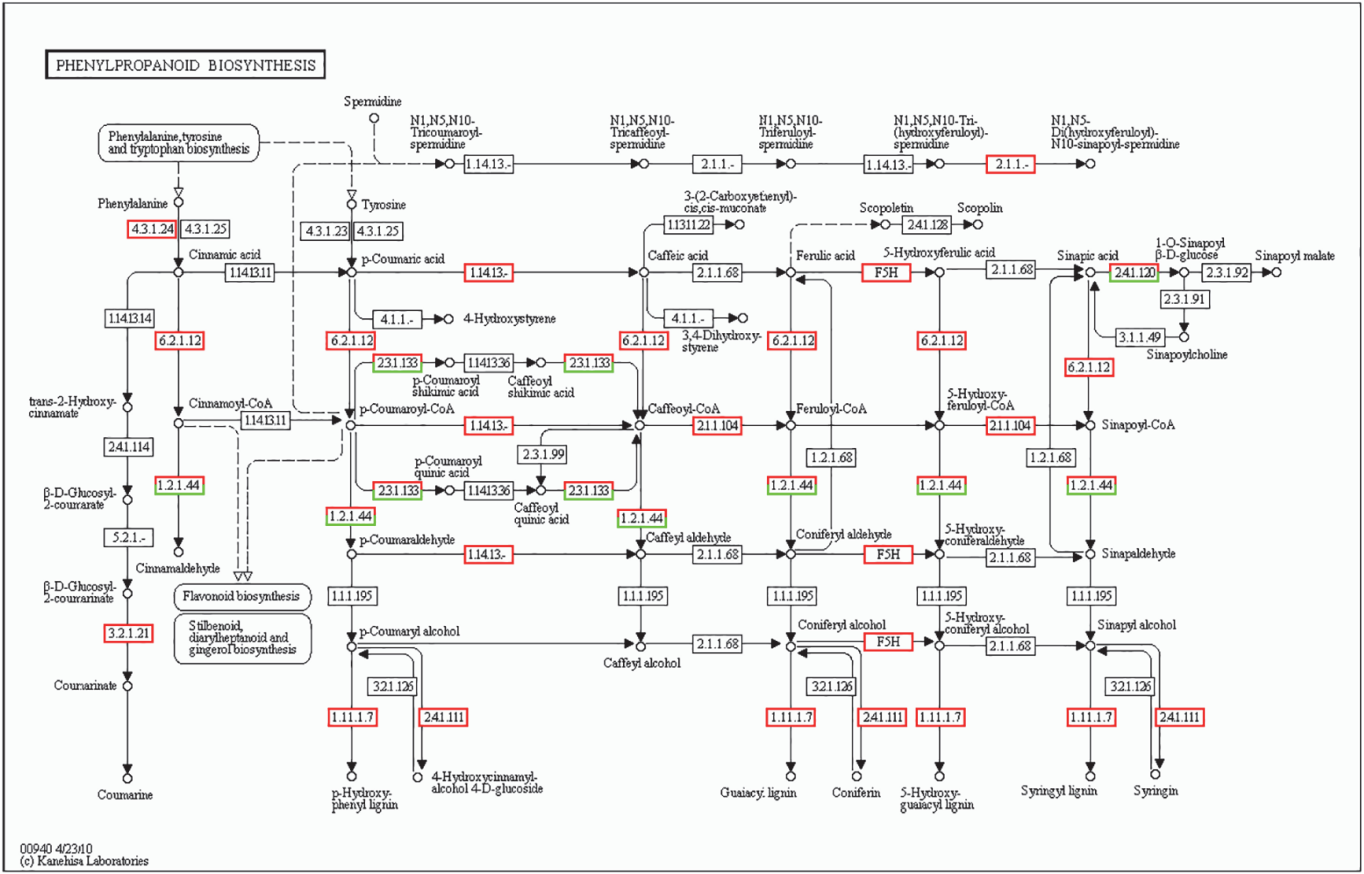
Change in gene expression of the phenylpropanoid biosynthesis pathway in foxtail millet 48h post-inoculation. Genes that up-regulated are marked with red borders while Genes that down-regulated are marked with green borders. Genes that did not change are marked with black borders.

The activation of phenylpropanoid metabolism, in which phenylalanine ammonia-lyase (PAL) catalyzes the first committed step of the core pathway, is a sign of plant responses to incompatible pathogens [35]. PAL is an enzyme involved in the biosynthesis of phenylpropanoids, such as monolignols, isoflavones, isoflavanones and stilbenes. Significant induction of PAL in the biosynthesis of phenylpropanoids and phenylpropanoid biosynthesis was observed in our study, similar to results observed in other plant-pathogen incompatible interactions. For example, upon inoculation with *Phaeoisariopsis personata*, PAL transcript levels increase in peanut plants [36]. Cytochrome P450 (CYP450) catalyzes 4-hydroxylation of cinnamic acid, which is an obligatory step in the biosynthesis of some phenolic compounds such as flavonoids, lignans, and stilbenes that are related to disease resistance in plants [37]. In this study, cytochrome P450 was involved in four pathways: biosynthesis of phenylpropanoids, phenylpropanoid biosynthesis, flavonoid biosynthesis, and flavone and flavonol biosynthesis. Therefore, the increase of PAL and CYP450 in our study could be related with millet resistance to infection by *U*. *setariae-italicae* (Fig 5).

### Validation of DGE data using qRT-PCR analysis

**Fig 6:**
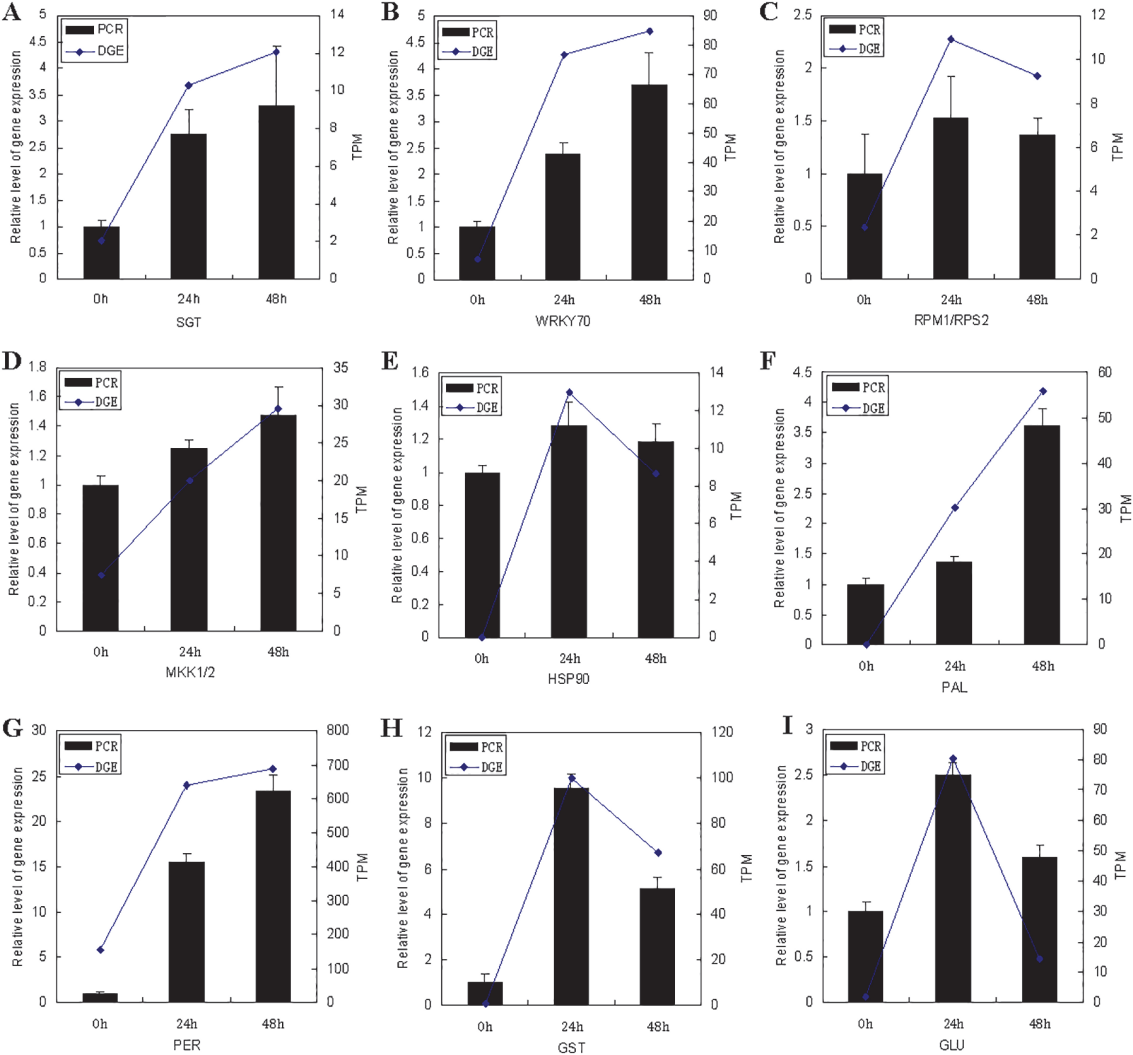
Quantitative RT-PCR validation of DGE analysis. TPM, transcription per million mapped reads. Relative quantification was carried out to measure changes in target gene expression in foxtail millet leaf samples relative to an endogenous reference gene; 18S rRNA was used as a reference gene. The X axis indicates the inoculation time. The Y axis indicates the fold change of target gene in qRT-PCR and TPM in DGE analysis. Bars represent standard errors of the means.

To validate the results of the DGE, nine genes were selected for confirmation using qRT-PCR (Fig 6). They included a suppressor of G2 allele of skp1 and related proteins (SGT), a WRKY transcription factor 70 (WRKY70), an NBS-LRR disease resistance protein (RPM1/RPS2), a mitogen-activated protein kinase kinase 6 (MKK1/2), a heat shock protein 90 (HSP90), a phenylalanine ammonia-lyase (PAL), a peroxidase (PER), a glutathione-S-transferase 24 (GST), and a β-1,3-glucanase (GLU). The expressions patterns of all nine genes demonstrated by qRT-PCR agreed well with DGE analysis. Among them, four genes (RPM1/RPS2, HSP90, GST, and GLU) were induced mainly after 24 h of post-inoculation, and five genes (SGT, WRKY70, MKK1/2, PAL, and PER) showed higher transcript levels in infected plants at 48 h of interaction.

**Fig 7:**
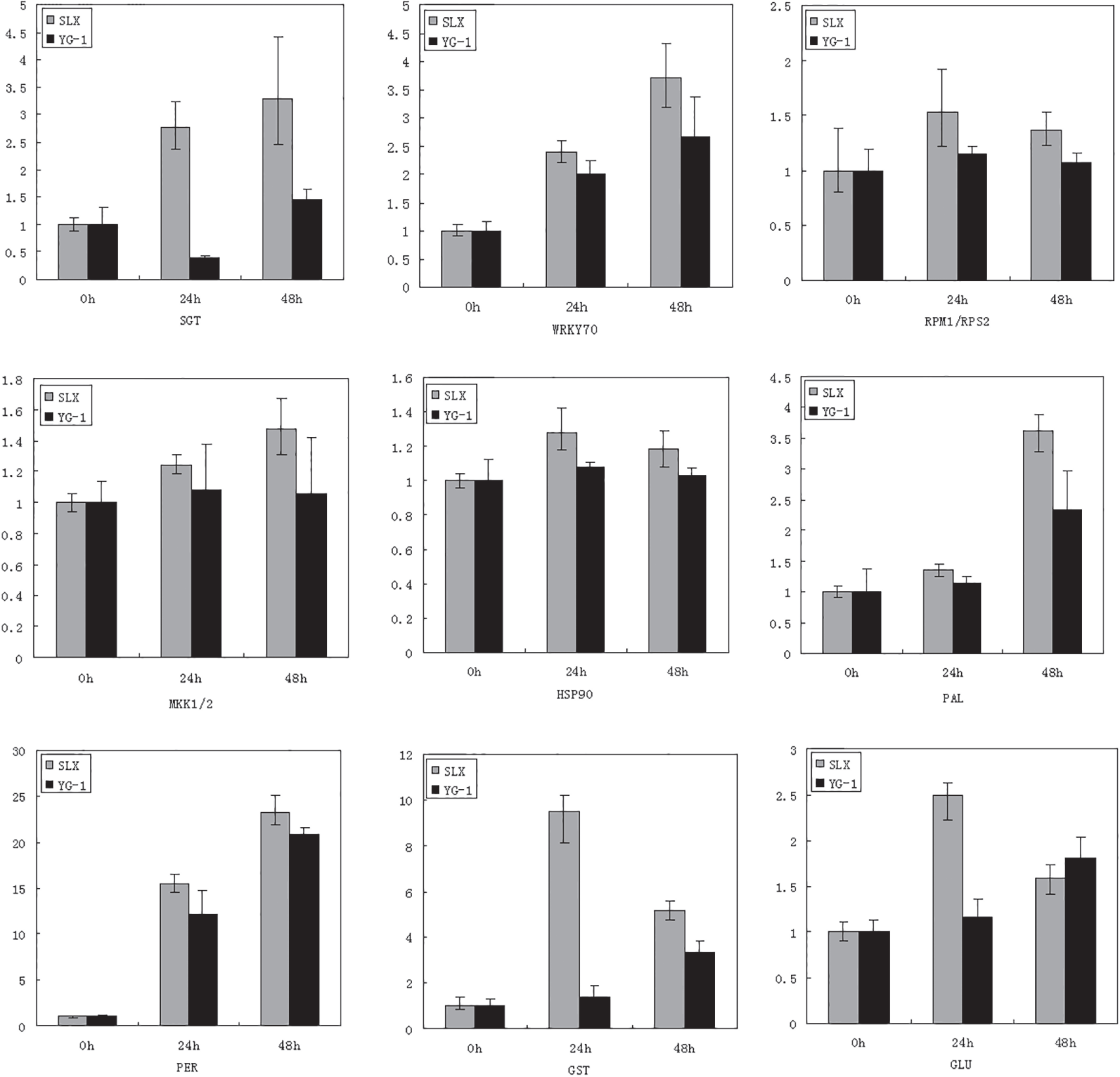
Gene expression level comparison between resistant cultivar Shilixiang and susceptible cultivar Yugu 1. The X axis indicates the inoculation time. The Y axis indicates the fold change of target gene in qRT-PCR. SLX represent resistant cultivar Shilixiang and YG-1 represent susceptible cultivar Yugu 1. Bars represent standard errors of the means.

Additionally, in order to determine whether the genes contribute to resistance to millet rust, expression levels of the genes were compared between the resistant cultivar Shilixiang and the susceptible cultivar Yugu-1. Results indicated that the genes can be induced in both cultivars, but with differences in their levels of gene expression: the expression level was higher in the resistant cultivar (Fig 7).

Induced expression of WRKY70, PAL and PER was continuous in both cultivars, but the expression level was higher in the resistant cultivar. Induced expression of SGT and MKK1/2 were continuous in Shilixiang, but in Yugu-1 they were down-regulated or up-regulated slightly. RPM1/RPS2, HSP90, GST, GLU were induced in Shilixiang and gene expression level reached peak 24 h post-inoculation, while the expression of these genes showed almost no change or fluctuation within a narrow range in Yugu-1.

## Supporting Information

S1 Fig *Uromyces setariae-italicae* inoculation experiment of two cultivars of foxtail millet in the field. (DOC)

S2 Fig Gene ontology (GO) classification of the DEGs. (DOC)

S1 Table Primer sequences used for qRT-PCR amplification. (DOC)

S2 Table Descriptive statistics for DGE sequencing. (DOC)

S3 Table Gene Ontology functional enrichment analysis for DEGs. (DOC)

S4 Table Selected partial genes that were up-regulated in foxtail millet after inoculation with the rust fungus *Uromyces setariae-italicae*. (DOC)
