# Supplementary material for: Differential Gene Expression in Foxtail Millet during Incompatible Interaction with Uromyces setariae-italicae
Source: PLoS One. 2015 Apr 17;10(4):e0123825. doi: 10.1371/journal.pone.0123825 (PMC4401669; doi:10.1371/journal.pone.0123825)
Supplement: S1 Fig — (DOC) [file pone.0123825.s001.doc]

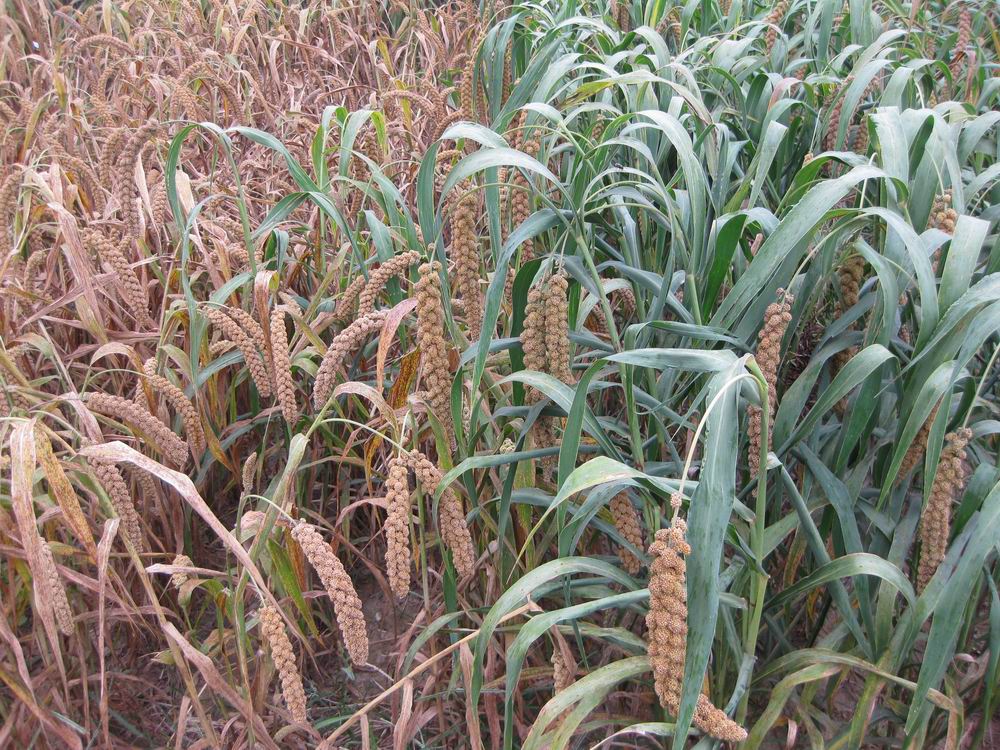


**S1 Fig. *Uromyces setariae-italicae* inoculation experiment of two cultivars of foxtail millet in the field.** The left is foxtail millet susceptible cultivar Yugu-1, and the right is resistant cultivar Shilixiang.
