# Supplementary material for: Differential Gene Expression in Foxtail Millet during Incompatible Interaction with Uromyces setariae-italicae
Source: PLoS One. 2015 Apr 17;10(4):e0123825. doi: 10.1371/journal.pone.0123825 (PMC4401669; doi:10.1371/journal.pone.0123825)
Supplement: S2 Fig — (DOC) [file pone.0123825.s002.doc]

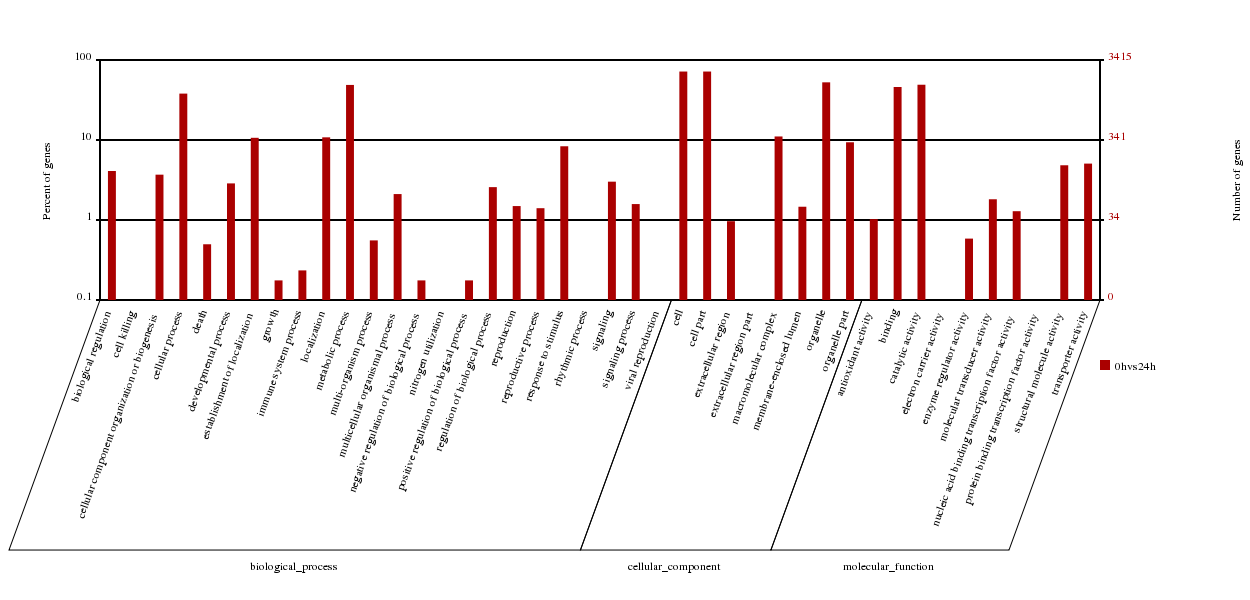


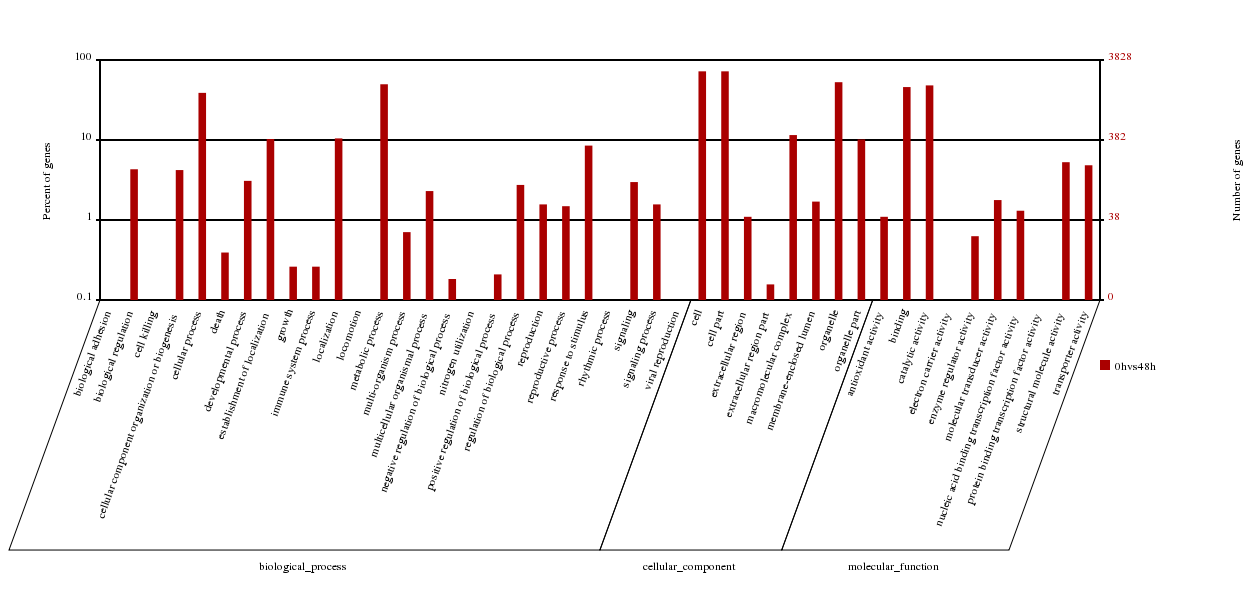


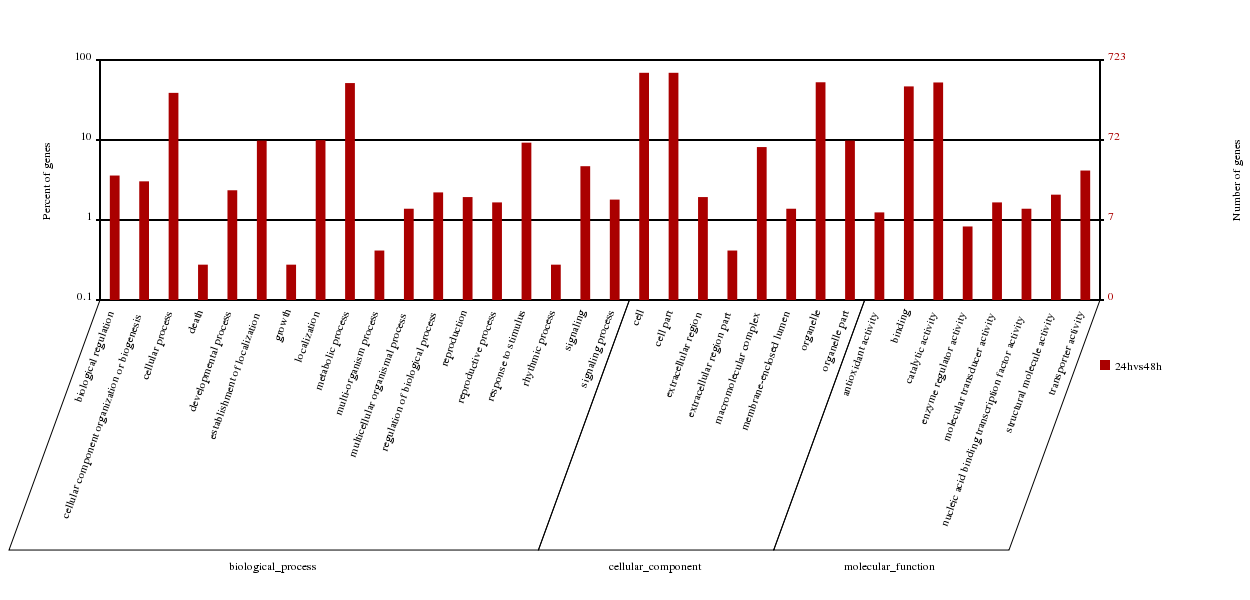


**S2 Fig. Gene ontology (GO) classification of the DEGs.** The results are summarized under the three main GO categories: biological process, cellular component and molecular function. The right y-axis indicates the number of genes in each category. The left y-axis indicates the percentage of a specific category of genes in the corresponding GO category.
