# Supplementary material for: Differential Gene Expression in Foxtail Millet during Incompatible Interaction with Uromyces setariae-italicae
Source: PLoS One. 2015 Apr 17;10(4):e0123825. doi: 10.1371/journal.pone.0123825 (PMC4401669; doi:10.1371/journal.pone.0123825)
Supplement: S1 Table — (DOC) [file pone.0123825.s003.doc]

**S1 Table. Primer sequences used for qRT-PCR amplification.**

| **Gene** | **Primer sequences (5’–3’)** | |
| --- | --- | --- |
|  | **Forward** | **Reverse** |
| SGT | TCCTGTGGCTGCTACTGTTGA | ACCACTGGCTGTGTATTCTCCAT |
| WRKY70 | AGCAGAGCGACGCGATGTA | CGTCTCGTGGCTCGTCCTT |
| RPM1/RPS2 | AGCAGCTCAAACTCTTGGATTCA | TTTCACTGTGATCCATTCATCGT |
| MKK1/2 | CAGAAGGAGAAGGTTGGATAAGCT | GCAGAAGGTGGTGGCTGATC |
| HSP90 | AGCAGTACGTGTGGGAGTCTCA | CCCCAGAGGTATCACGTGTGA |
| PAL | ATCGCCATGGCCTCCTACT | CTCTGCACGTGGTTGGTGAT |
| PER | AGCCCGTCGCAGTTCAAC | TGCCGGCCCCAATGT |
| GST | CCTCCGACGAGACAGTGAAGA | TCCTTGTTAGCGGCGAAGA |
| GLU | GCGCGCTGGATACCTTCAT | GGGATCCCCGCTCTTCTG |
