# Supplementary material for: Differential Gene Expression in Foxtail Millet during Incompatible Interaction with Uromyces setariae-italicae
Source: PLoS One. 2015 Apr 17;10(4):e0123825. doi: 10.1371/journal.pone.0123825 (PMC4401669; doi:10.1371/journal.pone.0123825)
Supplement: S2 Table — (DOC) [file pone.0123825.s004.doc]

**S2 Table. Descriptive statistics for DGE sequencing**.

| **Summary** |  | **0h** | **24h** | **48h** |
| --- | --- | --- | --- | --- |
| Raw Data | Total | 3627461 | 3652847 | 3514395 |
| Raw Data | Distinct Tag | 170420 | 220862 | 240923 |
| Clean Tag | Total number | 3397826 | 3394432 | 3238980 |
| Clean Tag | Distinct Tag number | 80668 | 105050 | 98892 |
| All Tag Mapping to Gene | Total number | 2048811 | 2083275 | 2024290 |
| All Tag Mapping to Gene | Total % of clean tag | 60.30% | 61.37% | 62.50% |
| All Tag Mapping to Gene | Distinct Tag number | 37497 | 48850 | 45176 |
| All Tag Mapping to Gene | Distinct Tag % of clean tag | 46.48% | 46.50% | 45.68% |
| Unambiguous Tag Mapping to Gene | Total number | 2040323 | 2075396 | 2016494 |
| Unambiguous Tag Mapping to Gene | Total % of clean tag | 60.05% | 61.14% | 62.26% |
| Unambiguous Tag Mapping to Gene | Distinct Tag number | 37317 | 48592 | 44957 |
| Unambiguous Tag Mapping to Gene | Distinct Tag % of clean tag | 46.26% | 46.26% | 45.46% |
| All Tag-mapped Genes | Number | 13806 | 16005 | 15426 |
| All Tag-mapped Genes | % of ref genes | 42.43% | 49.19% | 47.41% |
| Unambiguous Tag-mapped Genes | Number | 13709 | 15887 | 15322 |
| Unambiguous Tag-mapped Genes | % of ref genes | 42.13% | 48.83% | 47.09% |
| Unknown Tag | Total number | 1349015 | 1311157 | 1214690 |
| Unknown Tag | Total % of clean tag | 39.70% | 38.63% | 37.50% |
| Unknown Tag | Distinct Tag number | 43171 | 56200 | 53716 |
| Unknown Tag | Distinct Tag % of clean tag | 53.52% | 53.50% | 54.32% |
