# Supplementary material for: Differential Gene Expression in Foxtail Millet during Incompatible Interaction with Uromyces setariae-italicae
Source: PLoS One. 2015 Apr 17;10(4):e0123825. doi: 10.1371/journal.pone.0123825 (PMC4401669; doi:10.1371/journal.pone.0123825)
Supplement: S3 Table — (DOC) [file pone.0123825.s005.doc]

**S3 Table. Gene Ontology functional enrichment analysis for DEGs.**

| **GO term ID** | **Gene Ontology term** | **Cluster frequency** | **Genome frequency of use** | **Corrected P-value** |
| --- | --- | --- | --- | --- |
| **0h VS 24h** | | | | |
| cellular component | | | | |
| GO:0030529 | ribonucleoprotein complex | 197 of 2506 in the list | 421 of 13335 in the genome | 2.69e-38 |
| GO:0032991 | macromolecular complex | 378 of 2506 in the list | 1143 of 13335 in the genome | 1.36e-31 |
| GO:0044435 | plastid part | 157 of 2506 in the list | 534 of 13335 in the genome | 1.36e-07 |
| GO:0009536 | plastid | 321 of 2506 in the list | 1305 of 13335 in the genome | 3.75e-06 |
| GO:0009526 | plastid envelope | 77 of 2506 in the list | 227 of 13335 in the genome | 5.25e-06 |
| GO:0044422 | organelle part | 320 of 2506 in the list | 1310 of 13335 in the genome | 8.39e-06 |
| GO:0016020 | membrane | 505 of 2506 in the list | 2203 of 13335 in the genome | 8.73e-06 |
| GO:0044446 | intracellular organelle part | 289 of 2506 in the list | 1167 of 13335 in the genome | 1.05e-05 |
| GO:0009507 | chloroplast | 80 of 2506 in the list | 247 of 13335 in the genome | 2.71e-05 |
| GO:0044434 | chloroplast part | 77 of 2506 in the list | 237 of 13335 in the genome | 3.98e-05 |
| GO:0009579 | thylakoid | 76 of 2506 in the list | 239 of 13335 in the genome | 0.00012 |
| GO:0044425 | membrane part | 327 of 2506 in the list | 1395 of 13335 in the genome | 0.00039 |
| GO:0009534 | chloroplast thylakoid | 64 of 2506 in the list | 197 of 13335 in the genome | 0.00039 |
| GO:0031976 | plastid thylakoid | 66 of 2506 in the list | 206 of 13335 in the genome | 0.00047 |
| GO:0031984 | organelle subcompartment | 66 of 2506 in the list | 206 of 13335 in the genome | 0.00047 |
| GO:0031975 | envelope | 124 of 2506 in the list | 464 of 13335 in the genome | 0.00186 |
| GO:0031224 | intrinsic to membrane | 274 of 2506 in the list | 1165 of 13335 in the genome | 0.00216 |
| GO:0031967 | organelle envelope | 120 of 2506 in the list | 448 of 13335 in the genome | 0.00229 |
| GO:0044436 | thylakoid part | 38 of 2506 in the list | 108 of 13335 in the genome | 0.00589 |
| GO:0043234 | protein complex | 166 of 2506 in the list | 673 of 13335 in the genome | 0.00943 |
| GO:0031974 | membrane-enclosed lumen | 50 of 2506 in the list | 161 of 13335 in the genome | 0.01724 |
| GO:0005840 | ribosome | 28 of 2506 in the list | 76 of 13335 in the genome | 0.02409 |
| GO:0031981 | nuclear lumen | 47 of 2506 in the list | 153 of 13335 in the genome | 0.03611 |
| GO:0043233 | organelle lumen | 47 of 2506 in the list | 153 of 13335 in the genome | 0.03611 |
| GO:0070013 | intracellular organelle lumen | 47 of 2506 in the list | 153 of 13335 in the genome | 0.03611 |
| molecular function | | | | |
| GO:0005198 | structural molecule activity | 165 of 2564 in the list | 337 of 13346 in the genome | 3.26e-33 |
| GO:0003723 | RNA binding | 86 of 2564 in the list | 255 of 13346 in the genome | 8.49e-06 |
| GO:0008135 | translation factor activity, nucleic acid binding | 42 of 2564 in the list | 115 of 13346 in the genome | 0.00366 |
| GO:0016765 | transferase activity, transferring alkyl or aryl (other than methyl) groups | 40 of 2564 in the list | 110 of 13346 in the genome | 0.00665 |
| biological process | | | | |
| GO:0006520 | cellular amino acid metabolic process | 116 of 2156 in the list | 340 of 10662 in the genome | 6.19e-07 |
| GO:1901564 | organonitrogen compound metabolic process | 180 of 2156 in the list | 599 of 10662 in the genome | 1.95e-06 |
| GO:0009064 | glutamine family amino acid metabolic process | 26 of 2156 in the list | 44 of 10662 in the genome | 1.30e-05 |
| GO:0044281 | small molecule metabolic process | 348 of 2156 in the list | 1345 of 10662 in the genome | 3.35e-05 |
| GO:0071704 | organic substance metabolic process | 1228 of 2156 in the list | 5547 of 10662 in the genome | 0.00011 |
| GO:1901605 | alpha-amino acid metabolic process | 62 of 2156 in the list | 165 of 10662 in the genome | 0.00012 |
| GO:0043436 | oxoacid metabolic process | 180 of 2156 in the list | 636 of 10662 in the genome | 0.00022 |
| GO:0006082 | organic acid metabolic process | 180 of 2156 in the list | 637 of 10662 in the genome | 0.00025 |
| GO:0010467 | gene expression | 363 of 2156 in the list | 1439 of 10662 in the genome | 0.00030 |
| GO:0019752 | carboxylic acid metabolic process | 178 of 2156 in the list | 631 of 10662 in the genome | 0.00033 |
| GO:0044710 | single-organism metabolic process | 417 of 2156 in the list | 1710 of 10662 in the genome | 0.00188 |
| GO:1901575 | organic substance catabolic process | 153 of 2156 in the list | 554 of 10662 in the genome | 0.00815 |
| GO:0006412 | translation | 68 of 2156 in the list | 209 of 10662 in the genome | 0.01223 |
| GO:0009056 | catabolic process | 156 of 2156 in the list | 583 of 10662 in the genome | 0.04122 |
| **0h VS 48h** | | | | |
| cellular component | | | | |
| GO:0030529 | ribonucleoprotein complex | 233 of 2824 in the list | 421 of 13335 in the genome | 8.83e-53 |
| GO:0032991 | macromolecular complex | 441 of 2824 in the list | 1143 of 13335 in the genome | 4.66e-43 |
| GO:0044435 | plastid part | 189 of 2824 in the list | 534 of 13335 in the genome | 1.15e-12 |
| GO:0044422 | organelle part | 389 of 2824 in the list | 1310 of 13335 in the genome | 1.74e-12 |
| GO:0009536 | plastid | 380 of 2824 in the list | 1305 of 13335 in the genome | 8.50e-11 |
| GO:0044446 | intracellular organelle part | 345 of 2824 in the list | 1167 of 13335 in the genome | 1.38e-10 |
| GO:0009526 | plastid envelope | 90 of 2824 in the list | 227 of 13335 in the genome | 2.10e-08 |
| GO:0016020 | membrane | 573 of 2824 in the list | 2203 of 13335 in the genome | 2.20e-07 |
| GO:0009579 | thylakoid | 90 of 2824 in the list | 239 of 13335 in the genome | 4.63e-07 |
| GO:0009507 | chloroplast | 92 of 2824 in the list | 247 of 13335 in the genome | 5.89e-07 |
| GO:0031974 | membrane-enclosed lumen | 66 of 2824 in the list | 161 of 13335 in the genome | 1.23e-06 |
| GO:0044434 | chloroplast part | 88 of 2824 in the list | 237 of 13335 in the genome | 1.52e-06 |
| GO:0044428 | nuclear part | 71 of 2824 in the list | 183 of 13335 in the genome | 5.18e-06 |
| GO:000953 | chloroplast thylakoid | 75 of 2824 in the list | 197 of 13335 in the genome | 5.50e-06 |
| GO:0031981 | nuclear lumen | 62 of 2824 in the list | 153 of 13335 in the genome | 5.79e-06 |
| GO:0043233 | organelle lumen | 62 of 2824 in the list | 153 of 13335 in the genome | 5.79e-06 |
| GO:0070013 | intracellular organelle lumen | 62 of 2824 in the list | 153 of 13335 in the genome | 5.79e-06 |
| GO:0031976 | plastid thylakoid | 77 of 2824 in the list | 206 of 13335 in the genome | 9.04e-06 |
| GO:0031984 | organelle subcompartment | 77 of 2824 in the list | 206 of 13335 in the genome | 9.04e-06 |
| GO:0005634 | nucleus | 72 of 2824 in the list | 190 of 13335 in the genome | 1.27e-05 |
| GO:0031967 | organelle envelope | 140 of 2824 in the list | 448 of 13335 in the genome | 3.75e-05 |
| GO:0009532 | plastid stroma | 83 of 2824 in the list | 237 of 13335 in the genome | 7.36e-05 |
| GO:0031975 | envelope | 142 of 2824 in the list | 464 of 13335 in the genome | 0.00011 |
| GO:0044425 | membrane part | 366 of 2824 in the list | 1395 of 13335 in the genome | 0.00015 |
| GO:0031224 | intrinsic to membrane | 312 of 2824 in the list | 1165 of 13335 in the genome | 0.00015 |
| GO:0005840 | ribosome | 35 of 2824 in the list | 76 of 13335 in the genome | 0.00015 |
| GO:0044436 | thylakoid part | 45 of 2824 in the list | 108 of 13335 in the genome | 0.00016 |
| GO:0044391 | ribosomal subunit | 27 of 2824 in the list | 57 of 13335 in the genome | 0.00136 |
| GO:0009522 | photosystem I | 9 of 2824 in the list | 11 of 13335 in the genome | 0.00450 |
| GO:0043227 | membrane-bounded organelle | 1545 of 2824 in the list | 6863 of 13335 in the genome | 0.00812 |
| GO:0015934 | large ribosomal subunit | 13 of 2824 in the list | 21 of 13335 in the genome | 0.00888 |
| GO:0043231 | intracellular membrane-bounded organelle | 1526 of 2824 in the list | 6782 of 13335 in the genome | 0.01125 |
| GO:0012505 | endomembrane system | 44 of 2824 in the list | 122 of 13335 in the genome | 0.01549 |
| GO:0034357 | photosynthetic membrane | 33 of 2824 in the list | 85 of 13335 in the genome | 0.02242 |
| GO:0043234 | protein complex | 180 of 2824 in the list | 673 of 13335 in the genome | 0.03513 |
| molecular function | | | | |
| GO:0005198 | structural molecule activity | 202 of 2868 in the list | 337 of 13346 in the genome | 1.29e-51 |
| GO:0003723 | RNA binding | 105 of 2868 in the list | 255 of 13346 in the genome | 2.58e-10 |
| GO:0008135 | translation factor activity, nucleic acid binding | 47 of 2868 in the list | 115 of 13346 in the genome | 0.00075 |
| biological process | | | | |
| GO:0071704 | organic substance metabolic process | 1425 of 2444 in the list | 5547 of 10662 in the genome | 6.09e-10 |
| GO:0010467 | gene expression | 432 of 2444 in the list | 1439 of 10662 in the genome | 9.56e-09 |
| GO:0006520 | cellular amino acid metabolic process | 129 of 2444 in the list | 340 of 10662 in the genome | 1.40e-07 |
| GO:0044281 | small molecule metabolic process | 401 of 2444 in the list | 1345 of 10662 in the genome | 1.77e-07 |
| GO:0019752 | carboxylic acid metabolic process | 206 of 2444 in the list | 631 of 10662 in the genome | 4.14e-06 |
| GO:0022613 | ribonucleoprotein complex biogenesis | 44 of 2444 in the list | 85 of 10662 in the genome | 4.80e-06 |
| GO:0043436 | oxoacid metabolic process | 207 of 2444 in the list | 636 of 10662 in the genome | 5.02e-06 |
| GO:0006082 | organic acid metabolic process | 207 of 2444 in the list | 637 of 10662 in the genome | 5.79e-06 |
| GO:0044710 | single-organism metabolic process | 483 of 2444 in the list | 1710 of 10662 in the genome | 9.71e-06 |
| GO:1901575 | organic substance catabolic process | 178 of 2444 in the list | 554 of 10662 in the genome | 0.00016 |
| GO:1901564 | organonitrogen compound metabolic process | 189 of 2444 in the list | 599 of 10662 in the genome | 0.00029 |
| GO:0009056 | catabolic process | 184 of 2444 in the list | 583 of 10662 in the genome | 0.00040 |
| GO:0006412 | translation | 79 of 2444 in the list | 209 of 10662 in the genome | 0.00058 |
| GO:0008152 | metabolic process | 1884 of 2444 in the list | 7827 of 10662 in the genome | 0.00098 |
| GO:1901605 | alpha-amino acid metabolic process | 64 of 2444 in the list | 165 of 10662 in the genome | 0.00236 |
| GO:0009064 | glutamine family amino acid metabolic process | 24 of 2444 in the list | 44 of 10662 in the genome | 0.00419 |
| GO:0044085 | cellular component biogenesis | 72 of 2444 in the list | 195 of 10662 in the genome | 0.00476 |
| **24h VS 48h** | | | | |
| cellular component | | | | |
| GO:0044436 | thylakoid part | 24 of 508 in the list | 108 of 13335 in the genome | 1.43e-10 |
| GO:0034357 | photosynthetic membrane | 21 of 508 in the list | 85 of 13335 in the genome | 4.05e-10 |
| GO:0009521 | photosystem | 18 of 508 in the list | 70 of 13335 in the genome | 6.72e-09 |
| GO:0009522 | photosystem I | 8 of 508 in the list | 11 of 13335 in the genome | 5.39e-08 |
| GO:0009507 | chloroplast | 32 of 508 in the list | 247 of 13335 in the genome | 1.07e-07 |
| GO:0009579 | thylakoid | 31 of 508 in the list | 239 of 13335 in the genome | 1.90e-07 |
| GO:0044434 | chloroplast part | 28 of 508 in the list | 237 of 13335 in the genome | 8.81e-06 |
| GO:0044435 | plastid part | 46 of 508 in the list | 534 of 13335 in the genome | 1.59e-05 |
| GO:0009536 | plastid | 85 of 508 in the list | 1305 of 13335 in the genome | 4.08e-05 |
| GO:0009534 | chloroplast thylakoid | 22 of 508 in the list | 197 of 13335 in the genome | 0.00052 |
| GO:0031976 | plastid thylakoid | 22 of 508 in the list | 206 of 13335 in the genome | 0.00107 |
| GO:0031984 | organelle subcompartment | 22 of 508 in the list | 206 of 13335 in the genome | 0.00107 |
| GO:0009526 | plastid envelope | 22 of 508 in the list | 227 of 13335 in the genome | 0.00485 |
| GO:0009532 | plastid stroma | 22 of 508 in the list | 237 of 13335 in the genome | 0.00921 |
| GO:0009570 | chloroplast stroma | 6 of 508 in the list | 28 of 13335 in the genome | 0.04699 |
| molecular function | | | | |
| GO:0051002 | ligase activity, forming nitrogen-metal bonds | 3 of 543 in the list | 3 of 13346 in the genome | 0.01185 |
| GO:0051003 | ligase activity, forming nitrogen-metal bonds, forming coordination complexes | 3 of 543 in the list | 3 of 13346 in the genome | 0.01185 |
| GO:0046906 | tetrapyrrole binding | 5 of 543 in the list | 15 of 13346 in the genome | 0.04142 |
| biological process | | | | |
| GO:0006091 | generation of precursor metabolites and energy | 28 of 464 in the list | 303 of 10662 in the genome | 0.04740 |
