# Supplementary material for: Differential Gene Expression in Foxtail Millet during Incompatible Interaction with Uromyces setariae-italicae
Source: PLoS One. 2015 Apr 17;10(4):e0123825. doi: 10.1371/journal.pone.0123825 (PMC4401669; doi:10.1371/journal.pone.0123825)
Supplement: S4 Table — (DOC) [file pone.0123825.s006.doc]

**S4 Table. Selected partial genes that were up-regulated in foxtail millet after inoculation with the rust fungus *Uromyces setariae-italicae***.

| **Gene** | **Gene identifier** | **0 h** | **24 h** | **48 h** | **Annotation** |
| --- | --- | --- | --- | --- | --- |
| plant-pathogen interaction | | | | | |
| Unigene27316 | PR1 | 0 | 5.89 | 3.7 | Pathogenesis-related protein 1 (protein of unknown function) |
| Unigene25649 | SGT1 | 2.06 | 10.31 | 12.04 | Suppressor of G2 allele of skp1 and related proteins |
| Unigene29385 | ERP1 | 77.99 | 131.39 | 150.05 | Elicitor-responsive protein 1 |
| Unigene30953 | WRKY70 | 7.06 | 76.89 | 84.9 | WRKY transcription factor 70 |
| Unigene30025 | WRKY62 | 0 | 8.84 | 26.86 | WRKY transcription factor 62 |
| Unigene32120 | RPM1/RPS2 | 2.35 | 10.9 | 9.26 | NBS-LRR disease resistance protein family-3 |
| Unigene28439 | CDPK | 0 | 3.83 | 1.23 | Calcium-dependent protein kinase, isoform AK1 |
| Unigene16382 | STPK | 1.77 | 25.63 | 16.98 | Serine/threonine protein kinase |
| Unigene16373 | RLK | 7.06 | 56.27 | 33.96 | S-domain class receptor-like kinase3 |
| Unigene32432 | MEKK1 | 12.66 | 55.38 | 34.58 | Mitogen-activated protein kinase kinase kinase 2 |
| Unigene8872 | MKK1/2 | 7.36 | 20.03 | 29.64 | Mitogen-activated protein kinase kinase 6 |
| Unigene734 | MKK4/5 | 0 | 9.13 | 10.5 | Mitogen-activated protein kinase kinase 4 |
| Unigene27654 | HSP90 | 0 | 12.96 | 8.64 | Heat shock protein 90 |
| Unigene14926 | NPR1 | 18.54 | 67.76 | 74.41 | Regulatory protein NPR1 |
| Unigene2346 | WAKL22 | 0 | 35.65 | 29.33 | Wall-associated receptor kinase-like 22 |
| biosynthesis of phenylpropanoids/phenylpropanoid biosynthesis | | | | | |
| Unigene23762 | PAL | 0 | 30.05 | 55.88 | Phenylalanine ammonia-lyase |
| Unigene13560 | PER | 154.22 | 640.46 | 689.41 | Peroxidase |
| Unigene25719 | PER1 | 0 | 13.85 | 14.2 | Peroxidase 1 |
| Unigene16385 | CYP450 | 1.47 | 14.14 | 21.3 | Cytochrome P450 |
| Unigene31625 | CS | 0 | 11.19 | 1.54 | Chalcone synthase |
| Unigene29127 | PCT | 10.89 | 52.73 | 64.84 | 3-phosphoshikimate 1-carboxyvinyltransferase; EPSP-synthase |
| Unigene29701 | HXK7 | 0 | 24.16 | 22.54 | Hexokinase 7 |
| Unigene5711 | PP | 70.93 | 332.9 | 425.75 | P-protein |
| metabolism of xenobiotics by cytochrome P450/glutathione metabolism | | | | | |
| Unigene29832 | GST 24 | 0.59 | 99.87 | 67.31 | Glutathione S-transferase GST 24 |
| Unigene28548 | GSTU6 | 11.77 | 310.21 | 162.09 | Glutathione S-transferase GSTU6 |
| starch and sucrose metabolism | | | | | |
| Unigene5531 | GLU | 1.77 | 80.43 | 14.51 | β-1,3-glucanase |
| amino sugar and nucleotide sugar metabolism | | | | | |
| Unigene26400 | PR4 | 5 | 61.87 | 26.24 | Endochitinase PR4 |
| aminoacyl-tRNA biosynthesis | | | | | |
| Unigene11521 | TTS | 1.18 | 29.46 | 31.18 | Tyrosyl-tRNA synthetase |
| natural killer-cell-mediated cytotoxicity | | | | | |
| Unigene26274 | MPK3 | 37.67 | 131.1 | 108.98 | Mitogen-activated protein kinase 3 |
| ribosome | | | | | |
| Unigene13265 | RL15 | 1.18 | 48.02 | 50.02 | 60S ribosomal protein L15 |
| terpenoid backbone biosynthesis | | | | | |
| Unigene30017 | UPS | 1.47 | 75.12 | 95.4 | Undecaprenyl pyrophosphate synthetase |
| biosynthesis of terpenoids and steroids | | | | | |
| Unigene10999 | BBLP | 3.24 | 25.34 | 26.86 | Brassinosteroid biosynthesis-like protein |
| novel genes with unknown function | | | | | |
| Unigene22957 | Unknown | 0 | 5.89 | 29.95 | Unknown |
| Unigene3348 | Unknown | 0 | 13.85 | 25.32 | Unknown |
| Unigene30394 | Unknown | 0 | 19.74 | 25.01 | Unknown |
| Unigene19495 | Unknown | 0 | 26.81 | 32.11 | Unknown |
| Unigene25577 | Unknown | 0 | 24.16 | 23.16 | Unknown |
| Unigene17926 | Unknown | 0 | 216.24 | 57.73 | Unknown |
| Unigene19594 | Unknown | 4.12 | 410.38 | 499.54 | Unknown |
| Unigene19447 | Unknown | 0.59 | 32.7 | 32.11 | Unknown |
| Unigene28022 | Unknown | 1.18 | 55.97 | 50.63 | Unknown |
| Unigene23958 | Unknown | 5.89 | 232.73 | 205.31 | Unknown |
| Unigene14656 | Unknown | 0.59 | 21.51 | 35.2 | Unknown |
| Unigene23701 | Unknown | 2.35 | 79.25 | 57.43 | Unknown |
| Unigene20932 | Unknown | 2.65 | 88.97 | 221.67 | Unknown |
| Unigene26885 | Unknown | 5.3 | 158.79 | 338.69 | Unknown |
| Unigene26022 | Unknown | 2.94 | 86.61 | 35.51 | Unknown |
| Unigene21310 | Unknown | 1.18 | 34.76 | 45.08 | Unknown |
| Unigene25107 | Unknown | 2.35 | 49.79 | 68.23 | Unknown |
| Unigene28299 | Unknown | 12.36 | 195.61 | 536.9 | Unknown |
| Unigene19154 | Unknown | 0.59 | 7.95 | 16.05 | Unknown |
